# Robust Variable-Step Perturb-and-Observe Sliding Mode Controller for Grid-Connected Wind-Energy-Conversion Systems

**DOI:** 10.3390/e24050731

**Published:** 2022-05-20

**Authors:** Ilham Toumi, Billel Meghni, Oussama Hachana, Ahmad Taher Azar, Amira Boulmaiz, Amjad J. Humaidi, Ibraheem Kasim Ibraheem, Nashwa Ahmad Kamal, Quanmin Zhu, Giuseppe Fusco, Naglaa K. Bahgaat

**Affiliations:** 1 Department of Electronics and Telecommunications, Faculty of New Technologies of Computing and Communication, University of Ouargla, Ouargla 30000, Algeria; toumi.ilham@univ-ouargla.dz; 2 Algeria LSEM Laboratory, Department of Electrical Engineering, University Badji Mokhtar, Annaba 23000, Algeria; bilel.maghni@univ-annaba.dz; 3 Department of Drilling and Rig Mechanics, Faculty of Hydrocarbons, Renewable Energies and Earth and Universe Sciences, University of Ouargla, Ouargla 30000, Algeria; oussama.hachana@gmail.com; 4 College of Computer and Information Sciences, Prince Sultan University, Riyadh 11586, Saudi Arabia; 5 Faculty of Computers and Artificial Intelligence, Benha University, Benha 13518, Egypt; 6 Department of Electronics, University of Badji Mokhtar, Annaba 23000, Algeria; amira.boulmaiz@univ-annaba.org; 7 Department of Control and Systems Engineering, University of Technology, Baghdad 10001, Iraq; amjad.j.humaidi@uotechnology.edu.iq; 8 Department of Computer Techniques Engineering, Dijlah University College, Baghdad 10001, Iraq; ibraheemki@coeng.uobaghdad.edu.iq; 9 Faculty of Engineering, Cairo University, Giza 12613, Egypt; nashwa.ahmad.kamal@gmail.com; 10 Department of Engineering Design and Mathematics, Frenchy Campus Coldharbour Lane, University of the West of England, Bristol BS16 1QY, UK; quan.zhu@uwe.ac.uk; 11 Department of Electrical and Information Engineering, Università degli Studi di Cassino e del Lazio Meridionale, 03043 Cassino, Italy; fusco@unicas.it; 12 Department of Communications and Electronics Engineering, Faculty of Engineering, Canadian International College (CIC), Shiekh Zayed City, Egypt; naglaa_kamel@cic-cairo.com

**Keywords:** robust variable-step perturb and observe, normalization, second-order sliding mode controller, systematic step size, super-twisting algorithm

## Abstract

In order to extract efficient power generation, a wind turbine (WT) system requires an accurate maximum power point tracking (MPPT) technique. Therefore, a novel robust variable-step perturb-and-observe (RVS-P&O) algorithm was developed for the machine-side converter (MSC). The control strategy was applied on a WT based permanent-magnet synchronous generator (PMSG) to overcome the downsides of the currently published P&O MPPT methods. Particularly, two main points were involved. Firstly, a systematic step-size selection on the basis of power and speed measurement normalization was proposed; secondly, to obtain acceptable robustness for high and long wind-speed variations, a new correction to calculate the power variation was carried out. The grid-side converter (GSC) was controlled using a second-order sliding mode controller (SOSMC) with an adaptive-gain super-twisting algorithm (STA) to realize the high-quality seamless setting of power injected into the grid, a satisfactory power factor correction, a high harmonic performance of the AC source, and removal of the chatter effect compared to the traditional first-order sliding mode controller (FOSMC). Simulation results showed the superiority of the suggested RVS-P&O over the competing based P&O techniques. The RVS-P&O offered the WT an efficiency of 99.35%, which was an increase of 3.82% over the variable-step P&O algorithm. Indeed, the settling time was remarkably enhanced; it was 0.00794 s, which was better than for LS-P&O (0.0841 s), SS-P&O (0.1617 s), and VS-P&O (0.2224 s). Therefore, in terms of energy efficiency, as well as transient and steady-state response performances under various operating conditions, the RVS-P&O algorithm could be an accurate candidate for MPP online operation tracking.

## 1. Introduction

Global energy consumption is mostly covered by fossil fuels that have a detrimental effect on the natural environment [1]. The increasing demand for energy with the consideration of global warming and environmental pollution has pushed interesting development of renewable energies. The wind system as an energy source has demonstrated important progression with a considerable production rate and maintenance cost [2]. It is the fastest-growing source, with a growing average of 20% per year in the energy sector [3]. A wind turbine (WT) can be categorized as variable- or fixed-speed. In the first configuration, the variable-speed wind turbine (VSWT), to permanently reach the maximum power point (MPP), its speed is constantly varied depending on the wind-speed fluctuations [4]. Hence, several generator types can be used, and the PMSG remains an attractive solution “without gearbox” in onshore and offshore applications, as it provides many advantages, such as: high energy production, good power/weight ratio, better reliability, and a high capacity to maximize energy production [5,6]. In addition, a variable speed, PMSG, horizontal axis, and direct drive without gearboxes are features that provide a positive impact on a WT system’s mechanical framework design [5]. They permit the development of even larger VSWTs at greater heights.

In a machine-side converter (MSC), the VSWT should operate at the optimum speed during changes in wind speed to produce the maximum electrical power. This is realized by a fast and adequate MPPT algorithm. In order to enhance the dynamic performances, the MPPT techniques have recently gained considerable interest [7,8]. In the recent literature, there are three categories of MPPT algorithms; namely, the indirect power controller (IPC), direct power controller (DPC), and artificial intelligence (AI) [9,10].

The first category (IPC) involves the following techniques: optimal torque (OT) [11], power signal feedback (PSF) [12], tip speed ratio (TSR) [13], and sliding mode control (SMC) [14]. TSR-based MPPT is the simplest technique with a faster response time in which the wind speed data are recorded by means of anemometers. However, the availability of speed sensors increases the complexity of the wind power system, as well as the implementation and maintenance cost. In the OT and PSF techniques, prior knowledge of the turbine generator’s mathematical model is necessary to predetermine the PMSG speed, TSR, and torque constant. However, it is difficult to precisely follow the MPP under a lower wind speed due to the relativity between the tracking speed and generator inertia. The SMC technique has been widely proposed in the literature [15] and is simple to implement, but it generates the well-known phenomenon of chattering, in which high-frequency oscillations around the MPP occur in a steady state caused by the sign function nature [16,17].

Furthermore, artificial intelligence (AI)-based MPPT control techniques, such as FLC [10] and ANN [18], have been proposed to track the MPP well, but their industrial applications are limited. The standard FLC-based MPPT technique requires many precise guidelines in the controller design, such as the quantity of choices to be measured, as well as the determination of fuzzification, inferences, and defuzzification [10]. In addition, a larger data memory space implies much more execution time to obtain the optimum solution, which is a significant drawback for online applications. The ANN-based MPPT technique is an expert knowledge strategy that requires a huge amount of data under various operating conditions. It usually needs a formal method to define the optimal network layout and number of neurons to place in the hidden layer. Indeed, choosing the initial values of the network weights and setting the learning step are of important concern [19].

The third family (DPC) allows tracking of the MPP by controlling the power fluctuation given by the mechanical speed under the wind speed variation. This category comprises P&O [8], incremental conductance (INC) [20,21], and optimum relation-based (ORB) [11]. The conventional INC technique provides good results under constant wind-speed conditions [7]. Meanwhile, their performances are not ensured under sudden and faster wind-speed variations.

The P&O technique has been used effectively to follow the optimum rotor speed with interesting ease of implementation, which renders it the most common and applied algorithm in the literature [22]. It was developed in such a way to perturb the rotor speed at several steps and then observe the change in the extracted power until the power–speed curve slope becomes zero. The perturbation and observation actions are realized without using anemometers. The suitable choice of step size is the major concern of the P&O algorithm, as it directly affects the WECS performances [3,23]. The step size during the disturbance of the rotor speed can be fixed or variable. If a small step size is adopted by using the classical P&O algorithm, the tracking speed response becomes very slow, which causes more power losses [5]. Meanwhile, it shows small steady-state oscillations around the MPP. In contrast, a large step size engenders a faster tracking-speed response but with large steady-state oscillations that harm larger inertia WTs, and hence reduce the performance of the WECS [24,25].

To overcome the downsides of the fixed-step (FS)-P&O algorithms and efficiently achieve the optimum dynamic performance of a WECS, many modified P&O versions have been proposed [2,5]. They can be classified into two main groups: modified and adaptive P&O algorithms. By applying the modified P&O algorithms, the variable step (VS) sizes are attained by dividing the P/ω curve into several operating areas, with each one having a predefined step size based on a synthesized curve or ratio.

Adaptive (A)-P&O was presented in [26,27]. The step size was modified according to an objective function that relied upon various control variables and the wind speed. This method provided interesting results under uniform atmospheric conditions. However, the performances were reduced under a large random wind-speed variation when the P/ω curve included multiple peak points. In [3], the proposed algorithm combined the generation of adaptive step sizes with dividing the P/ω curve into several sections. The authors of [28,29] used a modified (M)-P&O algorithm based on the comparison of several P/ω curves and the sector’s intersection points. It employed a forward large step and a small step around the MPP. Meanwhile, the larger step induced oscillations at steady state with no structured relation to select the required step length and WT properties. The authors of [30] proposed a robust MPPT control scheme for a grid-connected PMSG-WT using a P&O-based nonlinear adaptive control. This approach used many assumptions that decreased the system efficiency caused by unwanted fluctuations around the MPP. A VS-P&O algorithm was developed in [2,22] in which the step size was determined by observing the distance between the operating point and the MPP in the P/ω curve. The authors subdivided the P/ω curve into modular operating sectors using predefined ratios. However, the performances of this approach remained poor under rapid climatic variations, as it needed to calculate a specific ratio at each wind-speed value. In [18,31], the authors suggested a hybrid P&O algorithm to eliminate the disadvantages of the conventional FS-P&O. Based on the error observation between the instantaneous and reference rotor speeds, the hybridized algorithm, while usually employing FLC, ANN, PSO, and ANFIS, etc., ensured the subdivision of the P/ω curve into several sectors. The simulation results showed the efficiency of the hybrid techniques in spite of the algorithm complexity.

Motivated by the above discussion, this paper presents a recently developed robust variable-step perturb-and-observe (RVS-P&O)-based MPPT algorithm to eliminate the drawbacks of the classical P&O technique, such as “slower time response, influence of the WT inertia and the step size selection concerns”. The control method proposed can realize stability in the system to maximize the power extraction in the WT system under rapid wind speed changes. Regarding the P/w curve, it is more appropriate to adjust the speed reference step as a function of the MPP error. Hence, it was proposed to adapt this step by a proportional factor to reach the MPP. To measure this action, the normalized power level was subdivided into a finite number of sectors. For each sector, the corresponding step size was determined as the optimum speed percentage. The objective of this work was to design the adaptive control in order to achieve the best performances of the MPPT operation. The main contributions of this paper can be summarized as outlined below.

- Normalization of the observation measurement and the speed variation allows the controller optimality to be maintained during the use of WTs with different dimensions. In addition to the normalization of the power measurement and the set-point speed increment:
- For more robustness, correction of the observation measurement was carried out by compensating for the wind speed effect;
- The RVS-P&O optimization strategy was based on subdividing the P/w curve into several modular operating sectors according to the distance in the ratio measurement between the actual and desired MPP;
- The RVS-P&O method improved the performance and the efficiency of VS-P&O algorithm variants while eliminating the drawbacks of the traditional FS-P&O ones;
- In terms of accelerated dynamic response capacity, the RVS-P&O algorithm tracked the MPP well during rapid climate variations, with a fast response time of 0.00794 s;
- The RVS-P&O approach enhanced the efficiency of the WECS by 3.82% compared to the conventional algorithms (FS-P&O and VS-P&O);
- The RVS-P&O algorithm showed a high level of stability with a small variation around the MPP, where the mean energy loss was estimated as 13.1826 W regardless of the operating conditions;
- The novel proposed approach was simple and easy to implement in practice;
- A DPC-SOSMC–STA controller was utilized in the grid-side converter (GSC) to obtain a smooth setting of the active and reactive power-quantity interchange between the generator and grid based on grid demand during realistic variable wind speeds.

To verify the performances of the proposed RVS-P&O algorithm, it was fairly compared to small step (SS)-P&O, large step (LS)-P&O, and VS-P&O techniques. The proposed algorithm was tested in different environmental conditions. This was on the basis of multiple data sets of wind speeds: gradual changes and experimental random variations. At the grid-side converter, a control strategy based on the DPC-SOSMC–super-twisting algorithm (STA) controller was utilized to realize the smooth setting of active and reactive power-quantity interchange between the generator and grid according to the real power request and variable wind speeds.

The rest of this paper consists of five sections that are organized as follows: Section 2 provides the mathematical modeling of the PMSG-based VSWT principal parts. The converter controller architecture is discussed in Section 3 and Section 4. Simulation tests using MATLAB/Simulink and comparison results are provided in Section 5 to validate the effectiveness of the proposed RVS-P&O algorithm under several operating scenarios of real wind-speed variation. Finally, Section 6 concludes by providing the obtained results and perspectives.

## 2. Mathematical Modeling of the WECS

**Figure 1:**
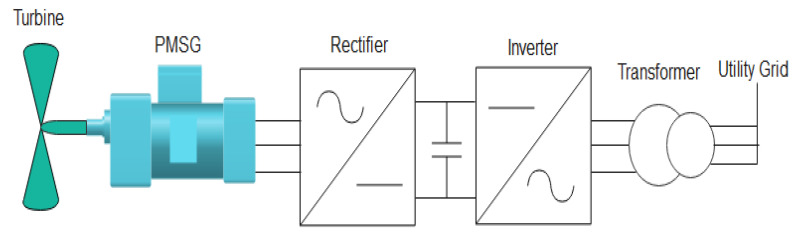
Configuration of the studied wind-generation system.

In order to establish the system control, the examined WECS is introduced in this section. Figure 1 depicts a representative topology of the considered WECS, which included a three-bladed turbine with a horizontal axis, with the rotor of the VSWT connected directly without a gearbox to the PMSG shaft [26]. The electronic power devices (two back-to-back AC/DC/AC IGBT bridges) supplied power from the used generator to the grid via a common DC bus [5,32].

### 2.1. Wind Turbine Model

A wind sail converts a quantity of air mass energy into movement; during the circulation of the wind in an active surface (S), the power of the air mass ($P_{\omega }$) is given by Equation (1) [33]:

(1) $$P_{\omega }=\frac{1}{2}\rho \cdot S\cdot v^{3}$$

It will be transmitted to the generator shaft as aerodynamic power or turbine power, as expressed by Equation (2) [34]:

(2) $$P_{k}=\frac{1}{2}\rho \cdot SC_{p}\left(\lambda ,\beta \right)\cdot V_{k}^{3}$$

where λ is the relation between the turbine angular speed and the wind speed. This denominates the tip speed ratio (TSR), and is given by Equation (3) [35]:

(3) $$\lambda =\frac{R\times \Omega_{k}}{V_{k}}$$

**Figure 2:**
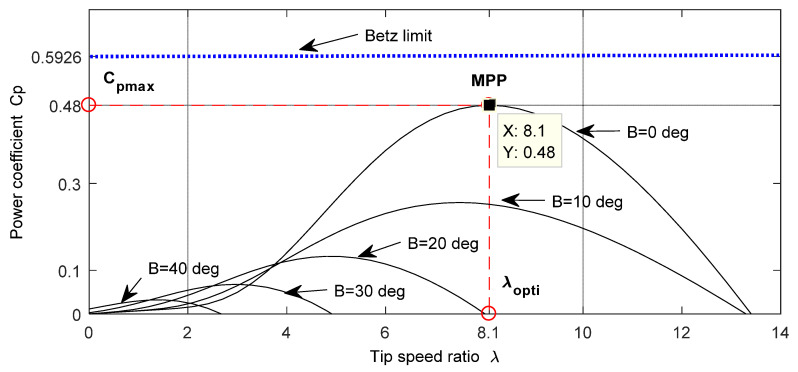
Model power coefficient $\left(C_{p}\right)$ with tip speed ratio (λ) curve.

The aerodynamic efficiency varies according to λ. In other words, the maximum $C_{p\;max}$ is reached when λ is optimal (${\;\lambda }_{opti}$). Figure 2 presents the resultant $C_{p}$ according to the λ variation when β is fixed [36].

### 2.2. PMSG Model

To enhance the controlling procedure of the electric generator’s dynamic performance, the model was based on the stator voltage within the Parck model, which is defined by using Equation (4) [37]:

(4) $$\{\begin{array}{c} V_{d}=R_{s}I_{d}+L_{d\;}\frac{dI_{d}}{dt}-\omega \;L_{q\;}\;I_{q\;}\;\\ V_{q}=R_{s}I_{q}+L_{q\;}{\frac{dI_{q}}{dt}}_{\;}+\omega \;(L_{\begin{array}{c} d\; \end{array}}\;I_{d\;}+\psi_{f})\; \end{array}$$

where $R_{s}$, $L_{d}$, $L_{q\;}$, and $\psi_{f}$ are given in Table A1 and Table A2 in Appendix A.

### 2.3. Grid Model

The grid model in the *d-q* plane is given by Equation (5) [4]:

(5) $$\{\begin{array}{c} V_{dg}=V_{di}-R_{g}I_{dg}-L_{dg}\frac{dI_{dg}}{dt}+L_{qg}w_{g}I_{qg}\;\\ V_{qg}=V_{qi}-R_{g}I_{qg}-L_{qg}\frac{dI_{qg}}{dt}-L_{dg}w_{g}I_{dg}\; \end{array}$$

where $\;R_{g}$, $L_{dg}$, and $L_{qg\;}$ are given in Table A3 in Appendix A.

## 3. Converter Controller Architecture

### 3.1. General Description

**Figure 3:**
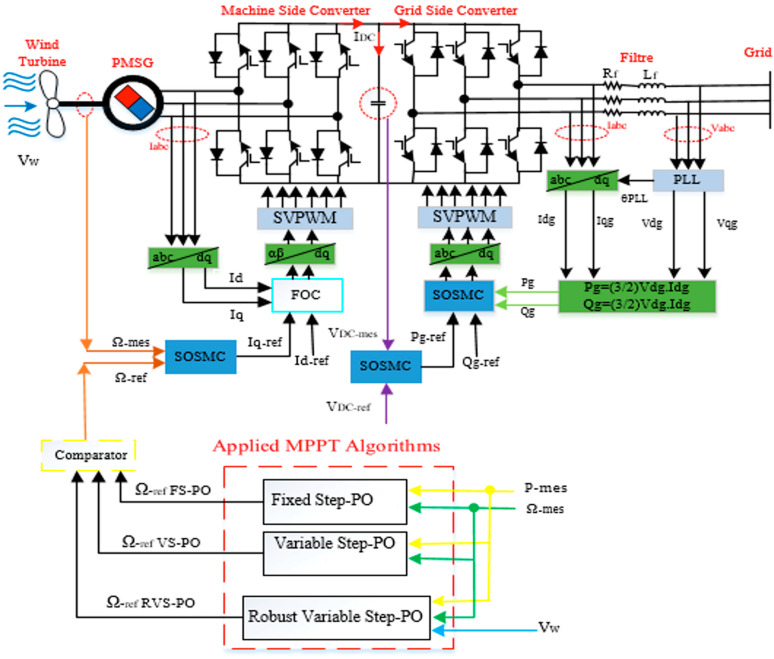
The complete control system description.

The energetic and environmental constraints of the WT-PMSG presented above required the application of a sophisticated supervision system and an adequate energy-management system. The control scheme is described in Figure 3, in which the control strategy was divided into two main parts: MSC and GSC.

- *Machine-Side Converter:* An advanced controller based on an RVS-P&O-based MPPT algorithm and the SOSMC were applied to control the PMSG speed and torque, thus extracting the MPP for each sampled wind speed value.
- *Grid-Side Converter:* While the wind speed fluctuated, the amplitude of the energy produced and the electrical frequency were constantly changing, which was not a perspective appropriate for grid integration. To resolve this problem, the GSC was usually employed to ensure the wind system’s connection to the electrical grid with better active and reactive powers. After that, the active and reactive power of the reference voltage generation was directly controlled by means of the DPC-SOSMC-STA-SVM strategy, unlike the traditional vector method.

### 3.2. Machine-Side Converter Controller

**Figure 4:**
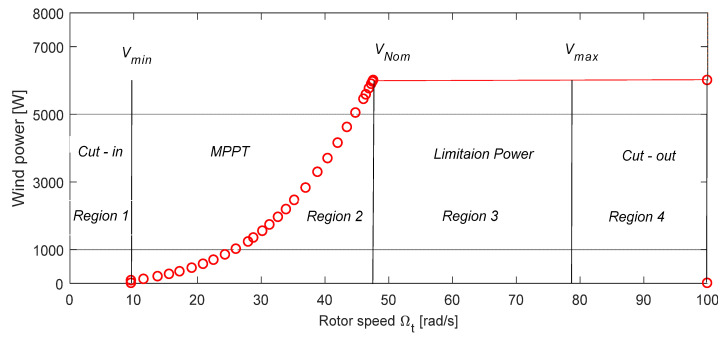
Power/speed curve showing the various operation regions of the VSWT.

A WT is usually characterized by the P/w curve showing the relationship between the rotor speed and the generated mechanical energy amount. Given the limits of $\left(v_{cut-in}\right)$ and $\left(v_{cut-out}\right)$ as shown in Figure 4, this work focused mainly on region (2) [31].

**Figure 5:**
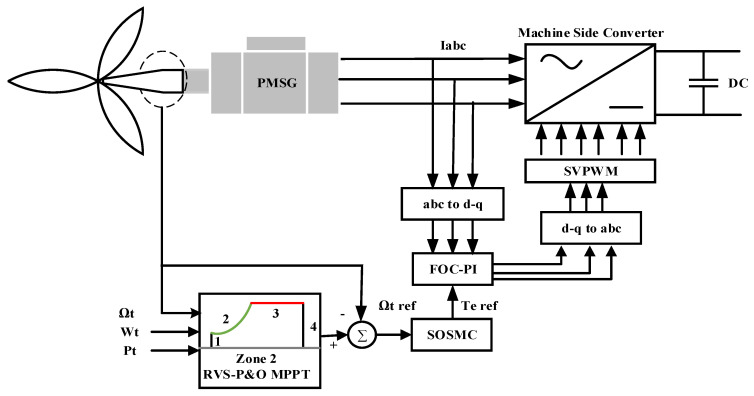
Block diagram of MSC contoller.

In this region (2), the maximum speed of the rotor could be reached by adjusting the electromagnetic torque to extract the highest mechanical power; this was done by keeping the power coefficient $\left(C_{p}\right)$ at the maximum $\left(C_{p\;max}\right)$. To achieve this goal, the field-oriented control (FOC) strategy was used to control the PMSG. It comprised two control loops, an external one for the speed and an internal one for the current, as depicted in Figure 5.

The RVS-P&O-MPPT-SOSMC algorithm was utilized in the first control loop to reach a reference optimal speed for each wind speed in order to generate an electromagnetic torque reference. The current control loop was exploited to control the stator currents of the *d-q* axis separately based on Equation (5). The PI controller was applied to adjust the three-phase currents by generating the commutation pulses by means of the space vector pulse-width modulation (SVPWM) technique [5].

### 3.3. Grid-Side Converter Controller

The utmost challenge in wind power generation is the inherently sporadic nature of the wind, which can deviate quickly [37]. Its intermittent availability is the main impediment to power quality and flow control. Wind-speed variations lead to a fluctuating injected power; therefore, the stability and power quality of the grid operation is affected. Consequently, the fluctuations in wind power should be reduced to prevent a degradation of the grid’s performance [38].

**Figure 6:**
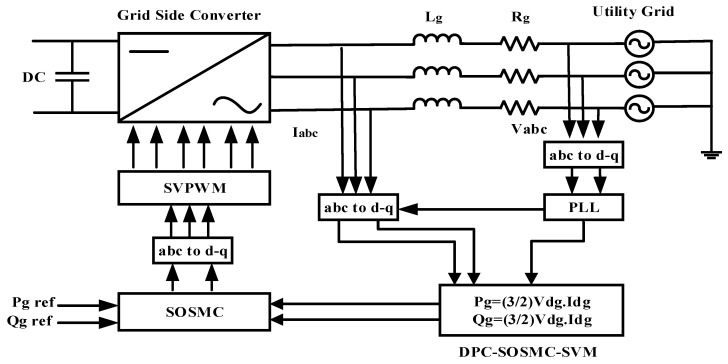
Block diagram of GSC-based DPC-SVM with SOSMC-STA controller.

For this reason, we proposed a GSC to provide and arrange the energy required by the user regardless of operational conditions [37]. For controlling the active and reactive power supplied into the electrical grid, a DPC-SVM-based SOSMC-STA was recommended at this stage. The schematic diagram of the GSC control approach is shown in Figure 6. In contrast to the traditional vector technique [38], the DPC-SVM-based SOSMC-STA approach provided the grid voltage directly to the GSC.

#### A. Higher Order SMC Based DPC-SVM Design

The first-order SMC engenders the chattering phenomena, which is a major inconvenience in practical operating implementation. To avoid such an issue, higher-order SMC application is a feasible solution that significantly reduces the multiple undesirable oscillations by maintaining the performances of the first-order controller [39,40].

The active and reactive grid powers are given by Equation (6) as follows:

(6) $$\{\begin{array}{c} P_{g}=\frac{3}{2}V_{dg}I_{dg}\\ Q_{g}=\frac{3}{2}V_{dg}I_{qg} \end{array}$$

In order to establish a null operating-power factor, the optimal reactive power was set to be $Q_{g\;ref}=0$, while the optimal active power $P_{g\;ref}$ depended on the grid requirement. The SOSMC block diagram is shown in Figure 6. The sliding surfaces of the active and reactive powers ($S_{P}$ and $S_{Q}$) were determined using Equation (7):

(7) $$\{\begin{array}{c} s_{P}=P_{gref}-P_{g}\\ \;s_{Q}=Q_{gref}-Q_{g} \end{array}$$

The first derivatives of the sliding surfaces are given by Equation (8):

(8) $$\{\begin{array}{c} {\dot{s}}_{P}=\;{\dot{P}}_{gref}-\frac{1.5\;V_{dg}}{L_{g}}\left(-V_{dg}-R_{g}I_{dg}+L_{g}w_{g}I_{qg}\right)-\frac{V_{id}}{L_{g}}\\ {\dot{s}}_{Q}=\;{\dot{Q}}_{gref}-\frac{1.5\;V_{qg}}{L_{g}}\left(-V_{qg}-R_{g}I_{qg}-L_{g}w_{g}I_{dg}\right)-\frac{V_{iq}}{L_{g}} \end{array}$$

Equation (9) gives the second derivative of both surfaces:

(9) $$\{\begin{array}{c} {\ddot{s}}_{P}={\dot{G}}_{p}-\frac{{\dot{V}}_{id}}{L_{g}}\\ {\ddot{s}}_{Q}={\dot{G}}_{Q}-\frac{{\dot{V}}_{iq}}{L_{g}} \end{array}$$

where $G_{P}$ and $G_{Q}$ are defined by Equation (10):

(10) $$\{\begin{array}{c} G_{P}={\dot{P}}_{gref}-\frac{1.5\;V_{dg}}{L_{g}}\left(-V_{dg}-R_{g}I_{dg}+L_{g}w_{g}I_{qg}\right)\\ G_{Q}={\dot{Q}}_{gref}-\frac{1.5\;V_{qg}}{L_{g}}\left(-V_{qg}-R_{g}I_{qg}-L_{g}w_{g}I_{dg}\right) \end{array}$$

The SOSMC defines two main parts, either for $V_{p}{}^{ref}$ or $V_{Q}{}^{ref}$, as given by Equation (11):

(11) $$\{\begin{array}{c} V_{P}{}^{ref}=V_{p}{}^{N}+V_{p}{}^{eq}\\ V_{Q}{}^{ref}=V_{Q}{}^{N}+V_{Q}{}^{eq} \end{array}$$

where $V^{N}$ is determined by Equation (12):

(12) $$\{\begin{array}{l} {\dot{w}}_{1}=-K\cdot sign\left(s_{P}\right)\\ w_{2\;}=-M\cdot \sqrt{\left|s_{P}\right|}sign\;\left(s_{P}\right)\\ V_{p}{}^{N}=w_{1}+w_{2}\\ V_{Q}{}^{N}=w_{1}+w_{2} \end{array}$$

The STA introduced by Levant [41] can be determined using Equation (13):

(13) $$\{\begin{array}{c} V_{p}{}^{ref}=V_{p}{}^{eq}-M\sqrt{\left|s_{P}\right|}sign\;\left(s_{P}\right)-K\int^{\;}sign\left(s_{P}\right)\;\;\;\\ V_{Q}{}^{ref}=V_{Q}{}^{eq}-M\sqrt{\left|s_{Q}\right|}sign\;\left(s_{Q}\right)-K\int^{\;}sign\left(s_{Q}\right) \end{array}$$

where K and M are unknown parameters to maintain the sliding manifolds’ convergence to zero in finite time [42]. Both parameters could be limited as determined by Equation (14):

(14) $$\{\begin{array}{c} K>\frac{C_{0}}{K_{m}}\;\;\;\;\;0<\rho <0.5\\ M^{2}\geq \frac{4C_{0}K_{M}\left(K-C_{0}\right)}{K_{m}{}^{2}K_{m}\left(K-C_{0}\right)}\;if\;\rho =0.5\; \end{array}$$

where $C_{0}$, $K_{m}$, and $K_{M}$ are positive constants.

## 4. MPPT-Based Control Algorithms

To enhance the overall efficiency of the WTs by capturing the highest energy output of the VSWT, an accurate MPPT algorithm should usually be implemented. Less-transient response oscillations, rapid dynamics, and a low design cost are the important requirements for an efficient MPPT technique. The VSWT is regulated to extract the highest generated power below the nominal wind speed. Therefore, to place the WT blades in front of the wind, the pitch angle should be zero. The MPP was determined by achieving the ideal values of $\lambda_{opt}$ and $C_{p\;opt}$, which were 8.1 and 0.48, respectively.

### 4.1. Classical P&O Algorithm

**Figure 7:**
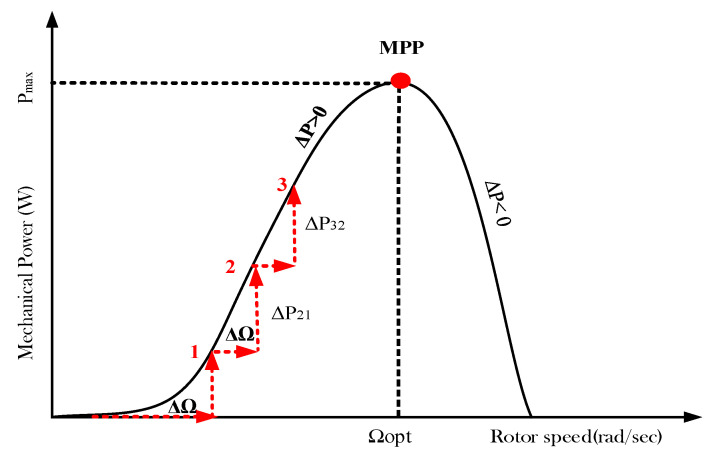
Working principal of the P&O-based MPPT technique.

The P&O algorithm is determined by the introduction of a small speed perturbation of (+ΔΩ−ref/+ΔΩ−ref), as illustrated in Figure 7. The effect of this disturbance is subsequently noticed in the PMSG output power.

A P&O algorithm is an iterative approach that needs just two sensors for sensing the power and the speed of the WT. Its operating principle, as depicted in Table 1, is based on perturbing the speed in small increments and comparing the power with that of the preceding perturbation cycle. If the perturbation leads to an increase (decrease) in wind power, the succeeding perturbation is made in the same (opposite) direction. In this manner, the MPP tracker incessantly seeks to find the maximum power location.

The behavior of the conventional P&O technique under varying climatic conditions was evaluated. In a basic analysis, this technique showed remarkable drawbacks, such as:

- The P&O algorithm step size was usually fixed and lacked any clarification regarding how it was determined;
- Through the observation of the P/w curve, it was more convenient to adjust the speed reference step according to the MPP error;
- The P&O algorithm was developed on the basis of a constant or slowly varying wind speed, which is not practical. In reality, the convergence rate is strongly affected by the rapid variation in the wind speed;
- The output power displayed several oscillations with a large magnitude permanently, even during fixed wind speeds.

To overcome these concerns, we proposed a robust variable-step P&O.

### 4.2. Proposed Robust Variable-Step P&O Algorithm

The RVS-P&O was based on the standardization of the generator speed and the mechanical power variables. Algorithm characteristic parameters are summarized in Table 2. A correction of the power-variation calculation was introduced by canceling the effect of wind disturbances.

#### 4.2.1. Power Normalization

To provide a systematic method for sizing the reference step size, a WT-PMSG system operating under the wind speed $v_{k}$ at instant k was considered. The maximum mechanical power $P_{k}^{max}$ is given by Equation (15):

(15) $$P_{k}^{max}=\frac{1}{2}\rho \cdot s\cdot v_{k}{}^{3}\cdot C_{p\;max}\;$$

To maintain the optimal controller dynamics with turbines of different sizes, a standardization of the power measurement and the set-point speed increment is suggested [43].

The normalized power $P_{k}^{N}$ is instantaneously defined as the ratio of the actual absorbed power to the maximum available one using Equation (16):

(16) $$P_{k}^{N}=\frac{P_{k}}{P_{k}^{max}}\times 100$$

#### 4.2.2. Speed Step Selection

If the speed reference step is taken to be constant, for considerable variation in wind speed, the controller will take more time to reach the MPP, as a nonadaptive step will provide the same action as that taken in a small variation in the wind speed case. Therefore, to avoid the slow reaction, an adaptation of this step size by a proportional amount to the correction signal to reach the MPP was proposed [42]. To subdivide the range of the normalized power in finite number of sectors (l=1…L), it is required to define (L−1) level as the delimiter. For that, let us consider a maximum power level in each sector, denoted by $P_{max}^{l}$ as a ratio ($\beta_{l}$) of the maximum mechanical actual power $P_{k}^{max}$, which is defined by means of Equation (17):

(17) $$P_{max}^{l}=\beta_{l}.P_{k}^{max}$$

where the ratio $\;\beta_{l}$ is in the range of [0,1], while l=1,…,L−1.

For each sector, the corresponding step size is defined by the weighting factor ($\alpha_{l\;}$) from the actual optimal speed $\;(\Omega_{k}^{opt})$ at instant k using Equation (18).

(18) $$\Delta \Omega_{k}^{ref}=\alpha_{l\;}\times \Omega_{k}^{opt}$$

**Figure 8:**
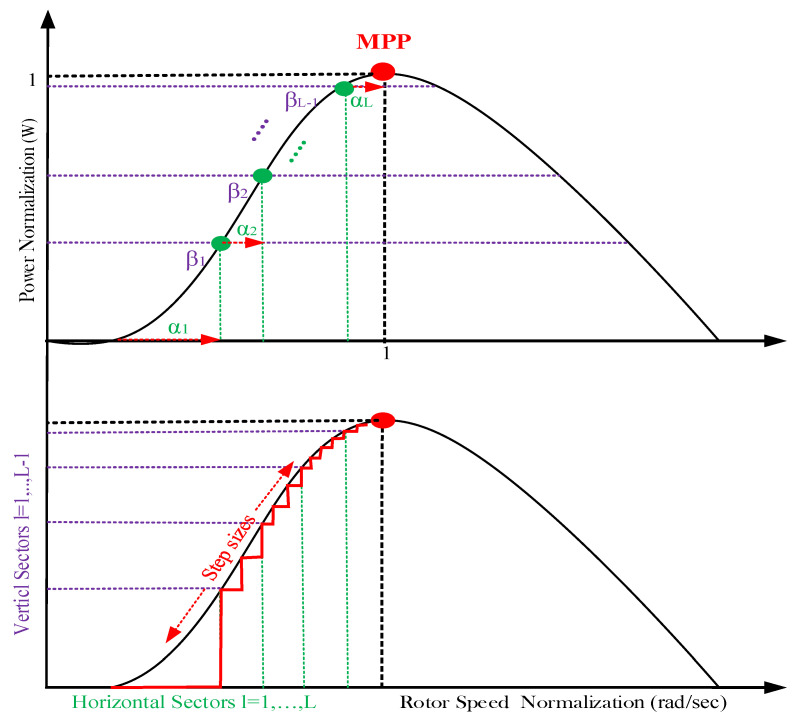
Operation principal of the RVS-P&O-based MPPT controller.

In addition, the normalized actual speed is defined by Equation (19):

(19) $$\Omega_{k}^{N}=\frac{\Omega_{k}}{\Omega_{k}^{opt}}\times 100$$

while the optimal speed ($\Omega_{k}^{opt}$) is given by Equation (20):

(20) $$\Omega_{k}^{opt}=\frac{\lambda_{opt}\times V_{k}}{R}$$

where l=1,…,L denotes the sector index and $\alpha_{l\;}$ is in the range of [0,1]. The weighting factor reflects the amount of the speed adjustment relative to the optimal speed. Since a fine adjustment is needed near the MPP, this factor should be decreased when moving from a sector to the upper one. Figure 8 shows an example of the normalized P/w curve with three modular operating sectors.

#### 4.2.3. Compensation for Wind-Speed Variation

The P&O algorithm is based essentially on the product sign of the power variation and the speed step increment. If positive, the speed reference step will be increased, and in the negative case, it will be decreased. The power variation also depends on the wind-speed variation, as it introduces a perturbation of the power variation $\Delta P_{k}$, and the algorithm will behave with less efficiency. This is one of the reasons we proposed the RVS-P&O strategy. It is necessary to eliminate this perturbation while taking into account only the part of $\Delta P_{k}$ induced by the speed adjustment in the previous step. This makes the control algorithm more robust against perturbations of the variation in wind speed. It is known that the power at instant k depends on the turbine speed and the wind speed as given by Equation (21):

(21) $$P_{k}=f\left(\Omega_{k},v_{k}\right)$$

Hence, the power variation at instant k is given by Equation (22):

(22) $$\Delta P_{k}=P_{k}\left(\Omega_{k},v_{k}\right)-P_{k}\left(\Omega_{k-1},v_{k-1}\right)$$

The development in the first order of Equation (22) is given by Equation (23):

(23) $$\Delta P_{k}≃f\left(\Omega_{k},v_{k-1}\right)+{\frac{\partial f}{\partial v}|}_{\left(\Omega_{k},v_{k-1}\right)}\Delta v_{k}-f\left(\Omega_{k-1},v_{k-1}\right)$$

The second term in Equation (23) represents the perturbation of the wind-speed variation. When the wind speed is constant, this term goes to zero. So, the corrected power variation $\Delta P_{k}^{\omega }$ without wind disturbance is determined by Equation (24):

(24) $$\Delta P_{k}^{\omega }=\Delta P_{k}-{\frac{\partial f}{\partial v}|}_{\left(\Omega_{k},v_{k-1}\right)}\Delta v_{k}$$

**Figure 9:**
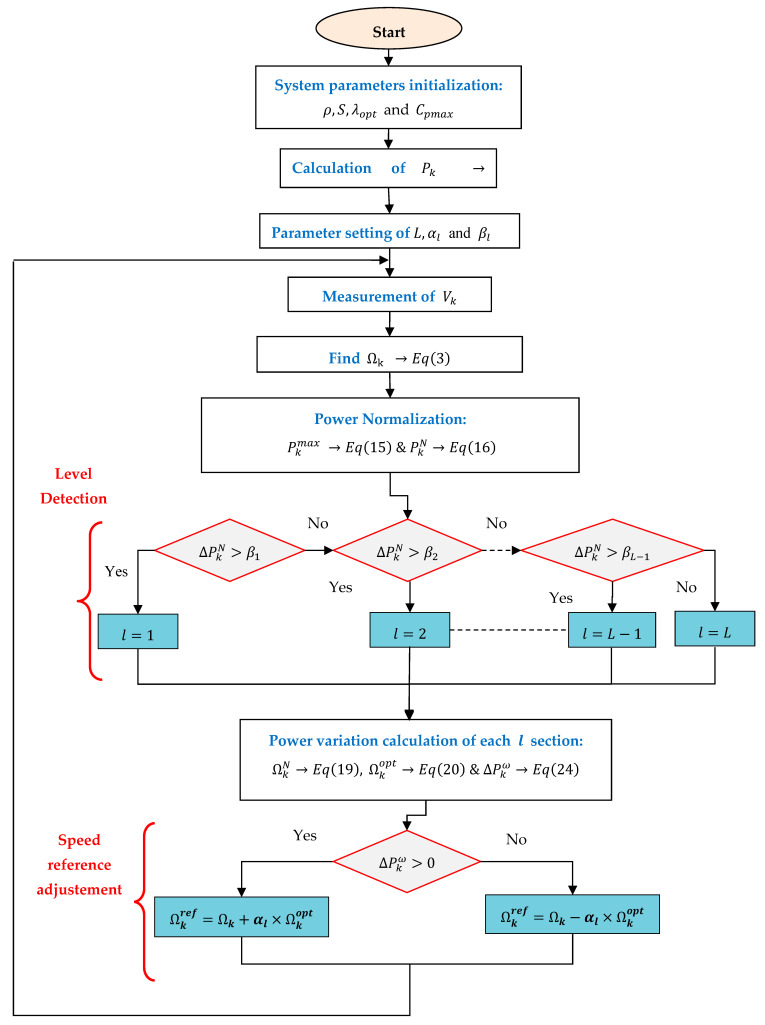
Detailed flowchart of the RVS-P&O-based MPPT technique.

Practically, $\Delta P_{k}$ is easily deduced through successively finding the error between the calculated powers ($P_{k}$ and $P_{k-1}$) at different instances. The RVS-P&O algorithm flowchart is illustrated in Figure 9.

It should be mentioned that the arbitrary parameter $\beta_{i}$ defines the corresponding sector size, since a coarse action should be taken when the operating point is located far from the MPP, and conversely. A fine step-size adjustment must be applied around the MPP, where the condition determined by Equation (24) is suggested:

(25) $$\;\beta_{l}=\{\begin{array}{c} 0.5\;\;\;\;\;\;\;\;\;\;\;\;\;\;\;\;\;\;\;\;\;\;\;l=1\\ 1.5\;\beta_{l-1}\;\;\;\;\;\;\;1<l<L \end{array}$$

This will ensure an initial fast response in the presence of perturbation at steady state.

## 5. Simulation Results and Discussion

To verify the effectiveness of the proposed algorithm, as well as its robustness compared to other existing MPPT algorithms, several simulations using MATLAB/Simulink were performed. This was done by using two different case studies of wind-speed profiles. Table A1, Table A2 and Table A3 in Appendix A provide the control parameters of the WT, PMSG, and grid, respectively. The organic ranking cycles (ORCs) and the overall efficiency of the WECS were calculated. The results are summarized in Table 3.

### 5.1. Gradual Variations in Wind Speed

**Figure 10:**
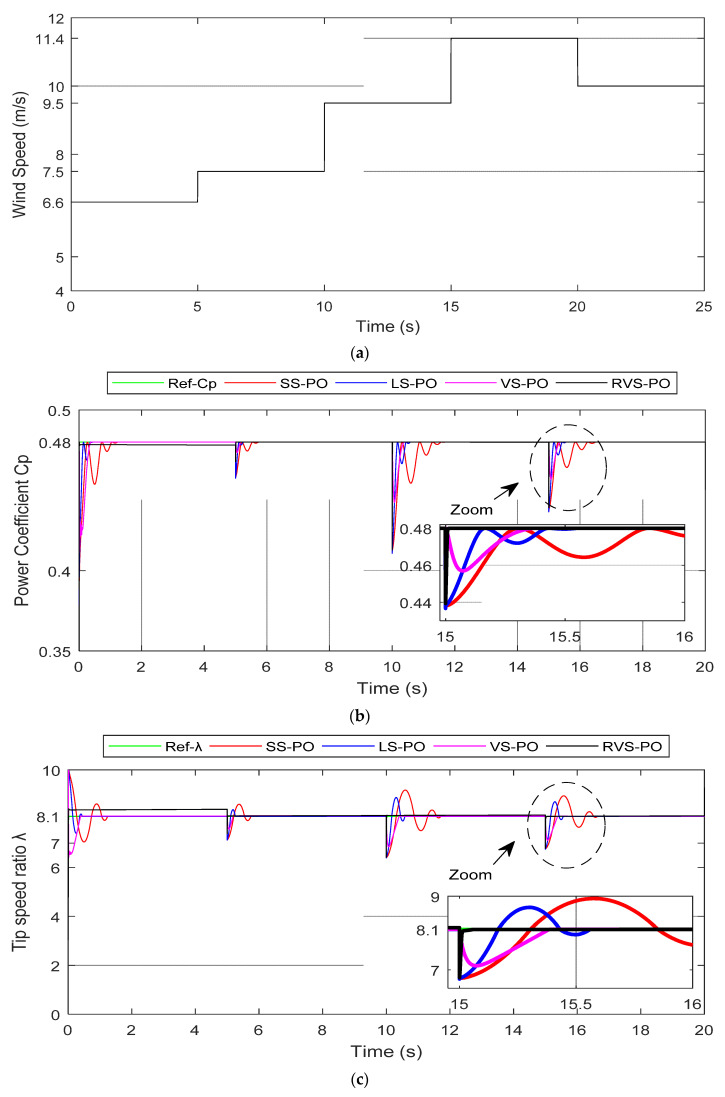

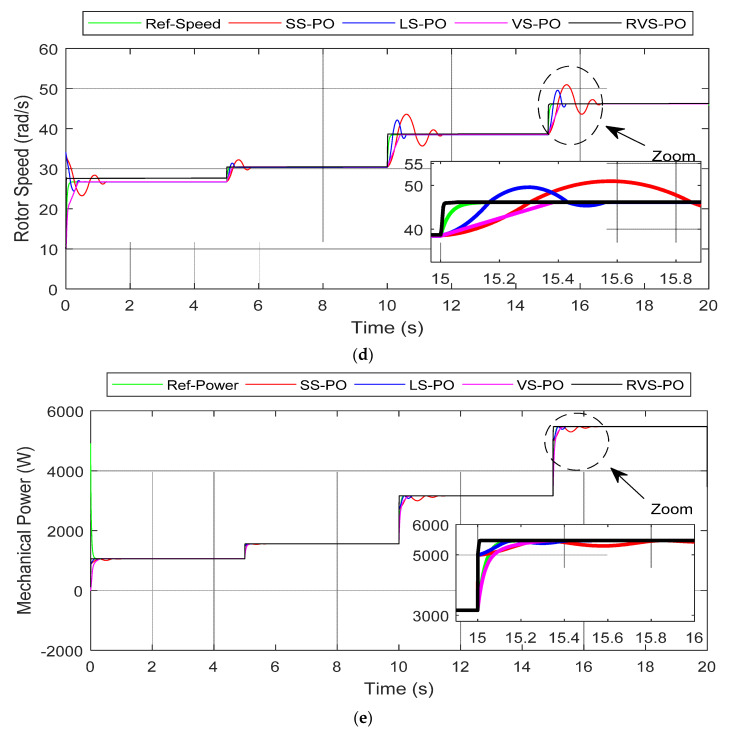
Machine-side results under gradual variations in wind speed. (**a**) Wind-speed profile; (**b**) Power coefficient; (**c**) Tip speed ratio; (**d**) Rotor speed; (**e**) Mechanical power.

Figure 10 depicts the machine-side results of four algorithms—SS-P&O, LS-P&O, VS-P&O, and RVS-P&O—under gradual variations in the wind speed. This was to well assess the transient and steady-state performances of the RVS-P&O as shown in Figure 10a. As can be observed, the predicted wind speed based on the step change profile was utilized to analyze the suggested P&O algorithms, in which the wind speed was varied by 6.6 m/s, 7.5 m/s, 9.5 m/s, 11.4 m/s, and 10 m/s every 5 s of samples. The obtained results were compared with the standard method (FS-P&O) and VS-P&O. The most important criteria to verify the effectiveness of the proposed technique were the optimal values of $C_{p}$ and *λ*. The behavior of the values is shown in Figure 10b,c. As shown in Figure 10b, the suggested algorithm (RVS-P&O) followed the ideal $C_{p}$ value faster than the SS-P&O, LS-P&O, and VS-P&O techniques, where the 5% settling time of 7.94 ms was compared to 161.7 ms, 84.1 ms, and 222.4 ms for the SS-P&O, LS-P&O, and VS-P&O techniques, respectively. In the transient response, during an abrupt variation in wind speed (9.5 m/s to 11.4 m/s) at 15 s, at this moment the SS-P&O and LS-P&O algorithms showed large oscillations around the MPP, with settling times of 1.5 s and 0.55 s, respectively. Meanwhile, RVS-P&O had an interesting settling time of 0.2 s compared to the VS-P&O algorithm, which had a time 0.37 s, as depicted in the zoomed part of Figure 10b.

The tip speed ratio was kept at the most optimal value (8.1) with all competing algorithms, as described in Figure 10c. Nevertheless, the RVS-PO effectively preserved the operation with an optimal TSR, and followed it with a lower settling time and without any overshooting as compared to the other algorithms during the fast wind change. At 15 s, the overshoot values of SS-P&O, LS-P&O, and VS-P&O were 8.939, 8.697, and 8.104, respectively. However, the RVS-P&O technique provided a better rapidity performance of 8.101, as depicted in the zoomed section of Figure 10c. Meanwhile, the rotor speed settling time was about 9.7 ms when using the RVS-P&O algorithm, as compared to the SS-P&O, LS-P&O and VS-P&O algorithms, which had times of 689.2, 330.4, and 310.2 ms, respectively, as it can be seen in the zoomed section of Figure 10d. Furthermore, it was clear that the RVS-P&O and VS-P&O algorithms had no remarkable overshoot on the tracking of the rotor speed compared to SS-P&O and LS-P&O.

Figure 10e depicts the mechanical power by means of the competing algorithms in order to verify the optimal power-extraction performances’ quality. The power oscillations of both algorithms, VS-P&O and LS-P&O, at steady state were lower around the extracted MPP. Meanwhile, the proposed RVS-P&O did not show any power oscillations for rapid variations in the wind speed. Simultaneously, the RVS-P&O algorithm took less time than the SS-P&O, LS-P&O, and VS-P&O algorithms to reach the new MPP under rapid fluctuations in the wind speed. For instance, during an abrupt variation of 9.5 m/s to 11.4 m/s at 15 s, the RVS-P&O algorithm required only 0.1 s, which was better than the time needed by the other algorithms (SS-P&O = 0.7 s, LS-P&O = 0.15 s, and VS-P&O = 0.3 s), as depicted in the zoomed part of Figure 10e. Therefore, RVS-P&O showed the best the power-extraction performances, as illustrated in Table 3.

### 5.2. Variable Fluctuations in Wind Speed

**Figure 11:**
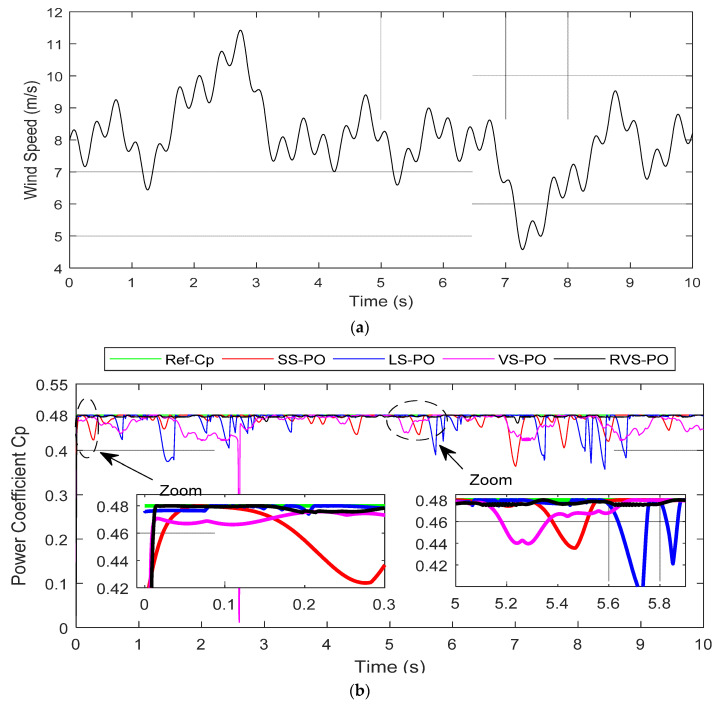

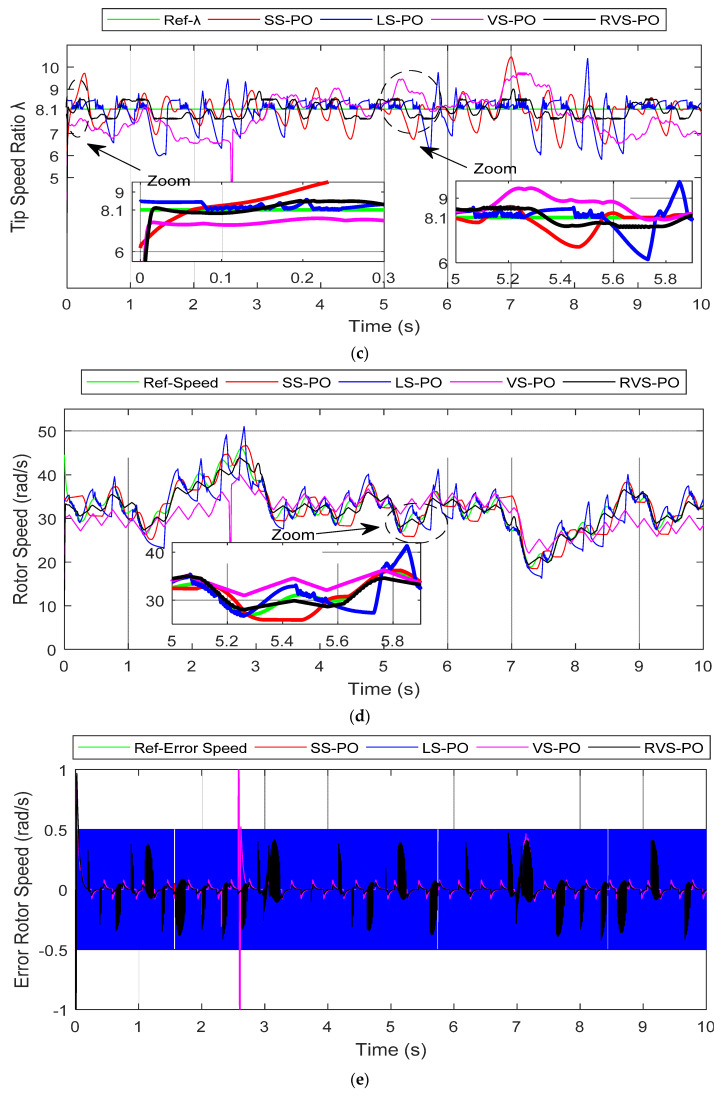

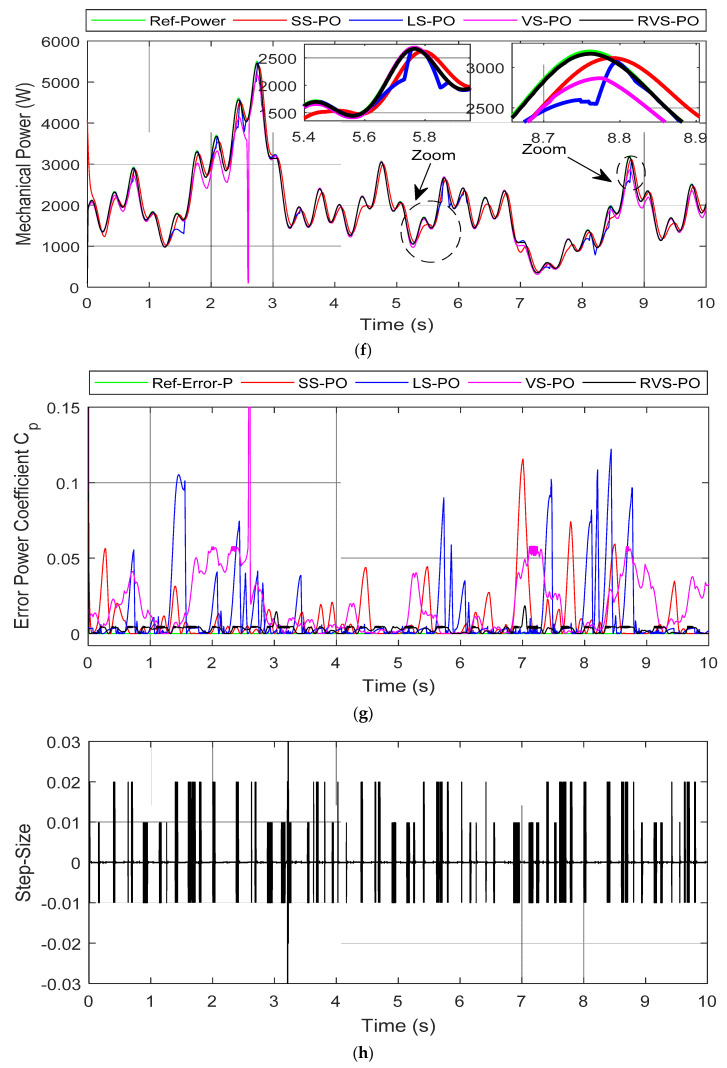
Machine-side results under variable fluctuations in wind speed. (**a**) Wind-speed profile; (**b**) Power coefficient; (**c**) Tip speed ratio; (**d**) Rotor speed; (**e**) Error rotor speed; (**f**) Mechanical power; (**g**) Extracted power error; (**h**) Step size.

Figure 11 demonstrates the machine-side results of the algorithms in competition under variable fluctuations in wind speed. In order to check the performance of the suggested RVS-P&O algorithm under variable environmental conditions, the system was simulated using a wind speed with an average value of 9 m/s, as shown in Figure 11a. The proposed RVS-P&O algorithm reached the optimal power coefficient ($C_{p}\;$= 0.48) more rapidly than the SS-P&O, LS-P&O, and VS-P&O algorithms, as depicted in the zoomed part of Figure 11b. It can be observed that the SS-P&O, LS-P&O, and VS-P&O algorithms were not able to efficiently track the MPP during these rapid operating conditions. Furthermore, they took more time to track the MPP due to the perturbation misdirection problem. In contrast, the proposed RVS-P&O sustained the optimal $C_{p}$ efficiently, with a mean value of 0.4770 during the 10 s wind speed variation. It can be mentioned that the mean $C_{p}$ values shown by the SS-P&O, LS-P&O, and VS-P&O algorithms were 0.4710, 0.4690, and 0.4616, respectively. The four algorithms preserved the optimal value of the TSR, as depicted in Figure 11c. However, RVS-P&O did not show any overshoot compared to the others, which presented relatively considerable ones. The rotor-speed tracking results are shown in Figure 11d. It is remarkable that the RVS-P&O was able to quickly regulate the generator speed under the rapid variation conditions, with very small ripples compared to SS-P&O, LS-P&O, and VS-P&O. Regarding the convergence aspect, the proposed algorithm quickly tracked the reference, with a lower speed error of 8.9757 × 10^−5^ rad/s compared to the competing algorithms, as displayed in Figure 11e and Table 3. An efficient speed tracking significantly increased the power-extraction quality, as the extracted power during 10 s in the same conditions was estimated at 2.0097 × 10^3^ W for RVS-P&O, while it was 1.9935 × 10^3^, 1.9679 × 10^3^, and 1.8868 × 10^3^ for SS-P&O, LS-P&O, and VS-P&O, respectively.

Figure 11f,g shows the RVS-P&O algorithm efficiency quality when tracking the MPP with small oscillations during random fluctuations in the wind speed. In addition, the waveforms of the mechanical power when using LS-P&O, SS-P&O, and VS-P&O showed some oscillations that affected the energy quality. This can be explained by their inability to track the MPP. The operating step sizes of the proposed RVS-P&O algorithm are depicted in Figure 11h.

### 5.3. Optimal Rotational Speed

**Figure 12:**
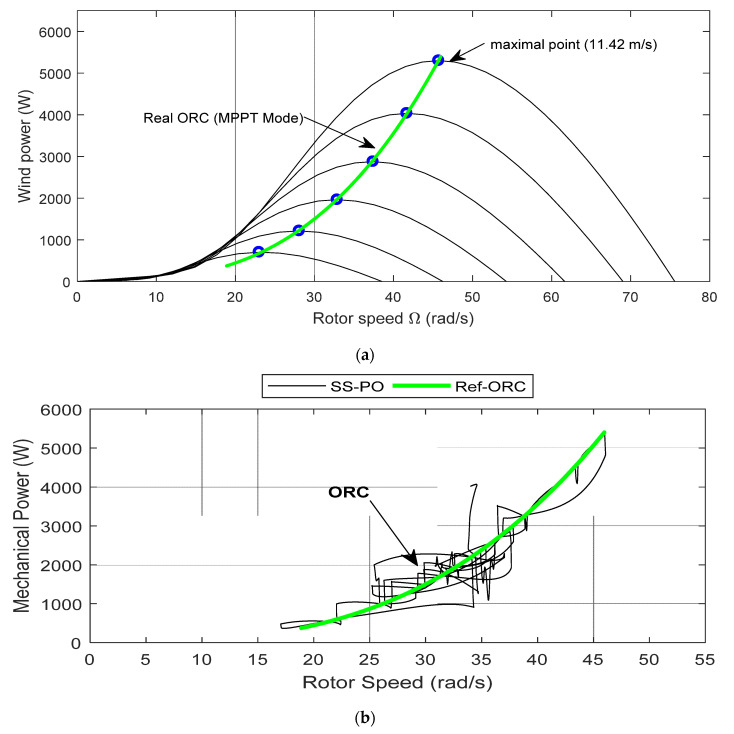

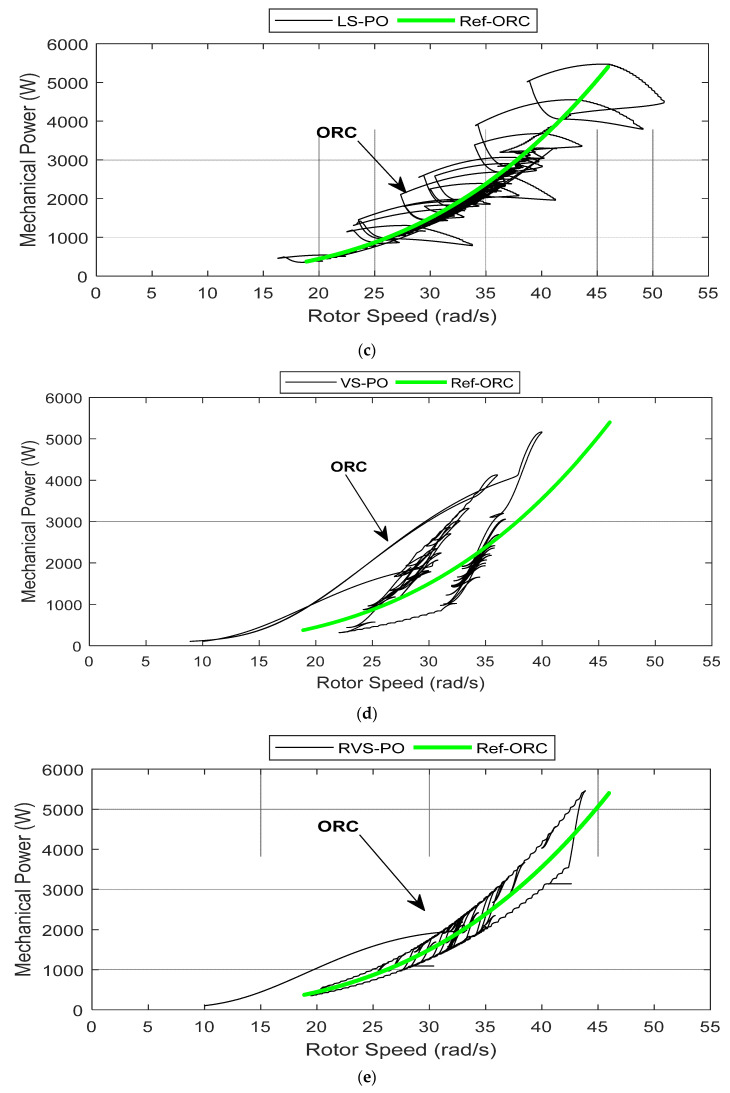
Optimal rotational-speed profile. (**a**) Real tracking of the optimal rotational speed ORC in region “II”; (**b**) ORC for SS-P&O; (**c**) ORC for LS-P&O; (**d**) ORC for VS-P&O; (**e**) ORC for RVS-P&O.

The organic ranking cycles also denominated the optimal rotational speed; evolutions by means of the four algorithms under variable fluctuations in wind speed are depicted in Figure 12. The wind energy system operated around the ORC while maintaining the MPP for each variation in the wind speed. Figure 12 illustrates the ORC profiles of the MPPT methods. The results were obtained by applying a mean wind profile of 11.55 m/s. It appears clearly that RVS-P&O was more efficient than the competing algorithms in the ORC smooth tracking, as shown in Figure 12e. The produced energy quality was better in terms of oscillation frequency and power loss, with an overall estimated efficiency of 99.35% by means of the proposed technique.

**Figure 13:**
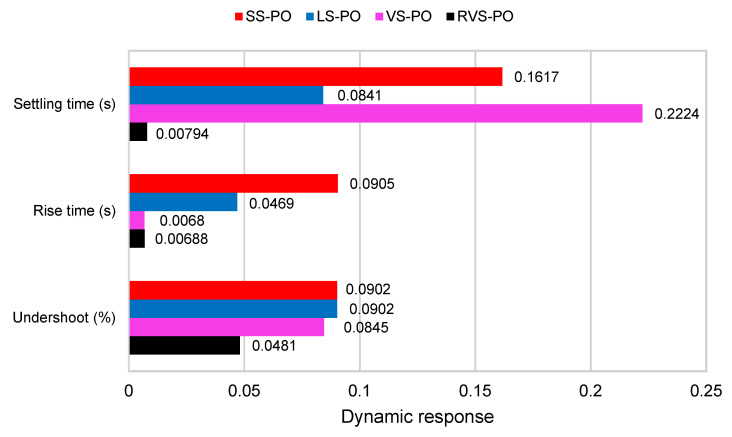
Dynamic response of the competing algorithms (SS-P&O, LS-P&O, VS-P&O, and RVS-P&O).

The dynamical behavior of the RVS-P&O-MPPT applied to the MSC was analyzed, as depicted in Figure 13, in terms of settling time, rise time, and undershoot. Whatever the instantaneous variations in the wind speed, the power extracted was the maximum value. The settling time (s) given by RVS-P&O was 0.00794; meanwhile, it was 0.2224 and 0.0841 for VS-P&O and LS-P&O, respectively. However, it was 0.1617 when using SS-P&O. Furthermore, the rise time was 0.0068 by means of RVS-P&O and VS-P&O, but it was 0.0496 and 0.0905 by using LS-P&O and SS-P&O, respectively. The undershoot (%) was 0.0481 when using RVS-P&O, which was better than for VS-P&O (0.0845), LS-P&O (0.0902), and SS-P&O (0.0902).

### 5.4. Grid-Side Converter DPC-SOSMC-STA Controller

**Figure 14:**
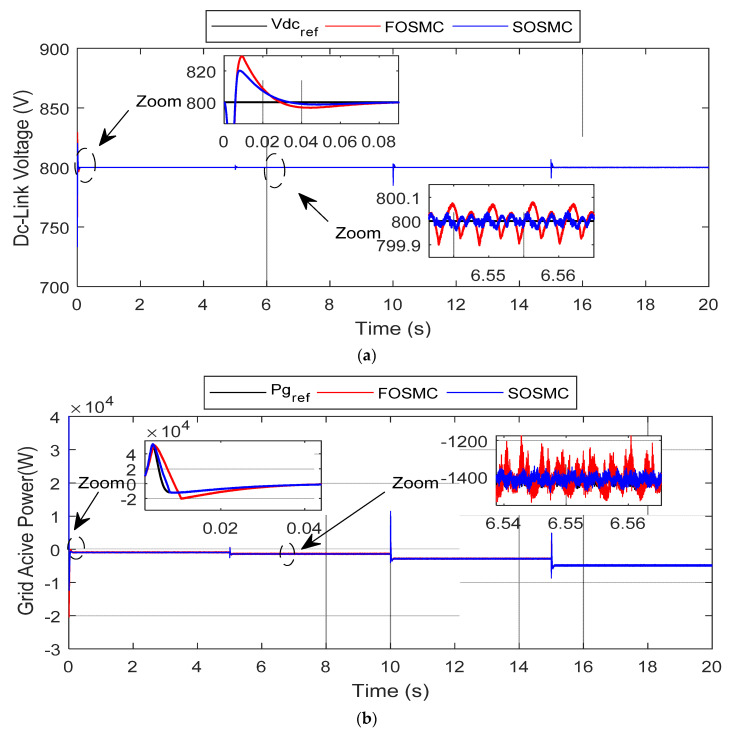

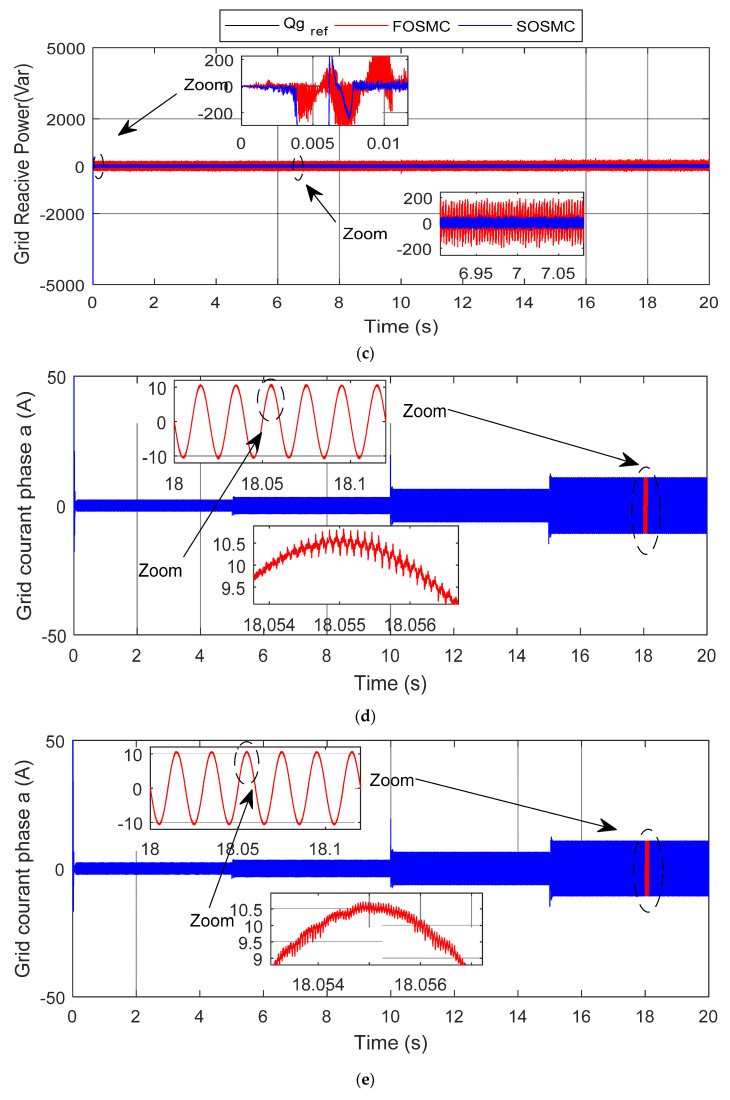

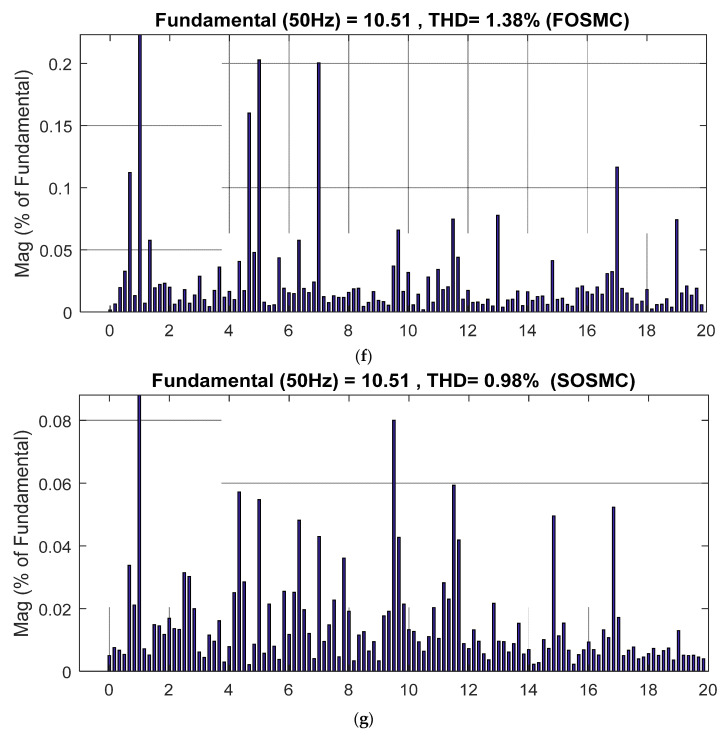
Grid-side results for FOSMC and SOSMC algorithms. (**a**) DC-link voltage; (**b**) Grid active power; (**c**) Grid reactive power; (**d**) Grid current phase “A” for FOSMC; (**e**) Grid current phase “A” for SOSMC; (**f**) THD for FOSMC algorithm; (**g**) THD for SOSMC algorithm.

When guaranteeing to supply the energy demanded, the quality of that energy is determined by the control tactics used in the regulation of parameters associated with the electrical grid. To achieve such an objective, a novel direct power control DPC–SVM that employed a nonlinear control SOSMC was developed. To evaluate the effectiveness of the proposed technique, a comparison with the FOSMC classical controller and the SOSMC was carried out; the results were validated by the harmonic analysis of each controller. The interchange of electric power between the PMSG and the grid is only assured if the DC bus is set to a constant value, regardless of the momentary variation in available power from the wind. The DC-link voltage of 800 V should be maintained around its nominal value by the machine-side converter, as depicted in Figure 14a. The electrical power injected into the grid was controlled by the DPC-SVM and two regulator types, as shown in Figure 14b,c. The required value could be accurately tracked by the FOSMC and SOSMC control units. However, there was a difference in the quality of the active and reactive powers. The simulation results revealed the superiority of the suggested regulator (SOSMC) based on the “super-adaptive convolution” algorithm that ensured high efficiency and a smooth desired slip path without the phenomenon of chatter or oscillations.

To illustrate the performance of the proposed control strategy (DPC–SVM) and the effectiveness of the SOSMC used in this work, an evaluation and a comparison with the conventional technique (FOSMC) was conducted. Figure 14d,e represent the grid injected current into phase A for both controllers. Furthermore, the THD of the current (phase A) was higher, at 1.38%. In Figure 14f, a distorted version of a highly unwanted current (phase A) can be seen during simulations in which the use of the FOSMC led to a poor quality of the grid’s electrical power. Through the smooth shape of the current, the superiority of the SOMSC was evident, as illustrated in Figure 14e. In addition, the decrease in the best current distortion reached 0.98%, as depicted in Figure 14g. The THD reduction, filtering, and the elimination of odd harmonics all showed considerable improvements [44,45]. Using the illustrated results, we deduced that the SOSMC approach attenuated 30% to 70% of the odd harmonics presented when using FOSMC.

## 6. Conclusions

To obtain an optimal and beneficial behavior in a wind turbine installation, an efficient MPPT technique to extract the wind power should be carried out. In this work, to eliminate the drawbacks of the existing conventional MPPT algorithms, particularly FS-P&O and VS-P&O, as they are highly used in current industrial applications, a new Robust variable-step P&O-based MPPT algorithm was proposed and validated under variable operating conditions of wind speed. The proposed RVS-P&O approach was based on the subdivision of the P/w curve into several horizontal modular operating sectors by comparing a newly synthesized ratio with another one related to the required power accuracy. To ensure an initial fast response in the presence of perturbations, the adjustment of arbitrary parameters ($\beta_{l}$) defined the corresponding sector size with a smooth alignment at steady state. In addition, to verify the performances of the proposed RVS-P&O algorithm, it was fairly compared to SS-P&O, LS-P&O, and VS-P&O techniques. The tracking-loss concern and the misdirection of the other techniques were avoided, and the step-size value was accurately estimated in each modular operating sector to reach the appropriate MPP.

In transient conditions, the proposed RVS-P&O algorithm reacted quickly to rapid fluctuations in wind speeds, with an interesting setting time of 7.94 ms and without any overshoot. In terms of steady-state stability, the RVS-P&O was more accurate than the competing algorithms. In both regimes, the proposed RVS-P&O algorithm combined lower oscillations with a power loss of 0.65% at 10 s variation, and a competitive tracking quality under limited speed fluctuations of 8.9757 ×10^−5^ rad/s. Furthermore, it provided an adeptly better quality of the extracted power during rapid changes in the wind speed, since the overall efficiency was 99.35% which was increased by 0.88%, 1.78%, and 3.82% compared to SS-P&O, LS-P&O, and VS-P&O, respectively. In fact, not only was the loss-of-tracking problem avoided, but the dynamic tracking performances also were improved in either transient or steady-state regimes under several operating conditions. The specified high-order SMC was built to manage the active and reactive powers exchanged between the generator and the grid in the GSC. The grid power values given by the SOSMC method, on the other hand, displayed smooth waveforms with acceptable tracking indices and low THD, as well as unwanted current distortion. In the case of the FOSMC control, the chattering phenomenon was ruled out.

## Figures and Tables

**Table 1:** Effect of wind-speed variation on the conventional P&O algorithm’s convergence rate.

	Operating Point is on the Left Side of $P_{max}$		Operating Point is on the Right Side of $P_{max}$	
	ΔΩ<0	ΔΩ>0	ΔΩ<0	ΔΩ>0
Increase in wind speed	Moves away to the left side of $P_{max}$	Converges toward the best $P_{max}$	Moves away to the left side of $P_{max}$	Moves away to the right side of $P_{max}$
Decrease in wind speed	Very slow convergence to reach $P_{max}$			

**Table 2:** Algorithm characteristic parameters.

Sector	$\beta_{L-1}$	$\alpha_{L}$
**l = 1**	0.6	0.03
**l = 2**	0.4	0.02
**l = 3**	0.01	0.01
**l = L**	0	0.0001

**Table 3:** Overall performance assessment of the competing algorithms under variable fluctuations in wind speed.

MPPT Method	Average Value			Average Error Value			Efficiency
	$\;P_{t\;\;}\left(w\right)$	$C_{p}$	λ	$\;P_{t\;\;\;}\left(w\right)$	$C_{p}$	$\Omega_{t}\;(\mathrm{rad}/s)$	η(%)
SS-P&O	1.9935 × 10^3^	0.4710	8.0282	30.9802	0.0090	6.5315 × 10^−4^	98.47
LS-P&O	1.9679 × 10^3^	0.4690	8.0029	49.1288	0.0110	0.0696	97.57
VS-P&O	1.8868 × 10^3^	0.4616	7.8862	88.2430	0.0184	2.3098 × 10^−4^	95.53
RVS-P&O	2.0097 × 10^3^	0.4770	8.0483	13.1826	0.0030	8.9757 × 10^−4^	99.35

## Data Availability

Not applicable.

## Nomenclature

Variables	
$C_{P}$	Coefficient power
F	Simplex
$f_{g}$	Grid frequency
$I_{d}$	d-axis current
$I_{\mathrm{dg}}$	Grid d-axis current
$I_{q}$	q-axis current
$I_{\mathrm{qg}}$	Grid q-axis current
K	First unknown gain
l	Sector index
$L_{d\;}$	d-axis inductance
$L_{q\;}$	q-axis inductance
M	Second unknown gain
m	Complex size
N	Normalization index
P	Number of independent complexes
$P_{g}$	Grid active power
$P_{t}$	Power of the air mass
$P_{k}$	Turbine power
$Q_{g}$	Grid reactive power
$R_{s}$	Stator resistance
S	Surface
$S_{P}$	Sliding surface of the active power
$s_{Q}$	Sliding surface of the reactive power
$T_{e}$	Electromagnetic torque
$\alpha_{L\;}$	Weighting factor
V	Wind speed
$V_{d}$	d-axis voltage
$V_{\mathrm{dg}}$	Grid d-axis voltage
$V_{\mathrm{di}}$	Inverter d-axis voltage
$V_{q}$	q-axis voltage
$V_{\mathrm{qg}}$	Grid q-axis voltage
$V_{\mathrm{qi}}$	Inverter q-axis voltage
W	Selection factor
Subscripts and superscripts	
d	stator axis
e	Electromagnetic
f	Flux
g	Grid
i	Element (solution)
j	Point index
k	Complex index
max	Maximum
mes	Measure
opti	Optimum
p	Power
q	Stator axis
ref	Reference
s	Stator
Greek letters	
α	Number of iteration for each simplex
	Blade pitch angle
λ	Tip speed ratio
ρ	Air density
τ	Number of offspring
ω	Electric pulsation
$\psi_{f}$.	Magnetic flux
Abbreviations	
AC	Alternating current
AI	Artificial intelligence
ANFIS	Adaptive neuro fuzzy inference system
ANN	Artificial neural network
DC	Firect current
DFIG	Doublyfed induction generator
DPC	Direct power control
FLC	Fuzzy logic control
FOSMC	First-order sliding mode controller
FOC	Field-oriented control
FS	Fixed step
GSC	Grid-side converter
INC	Incremental conductance
IPC	Indirect power controller
LS	Large step
MPPT	Maximum power point tracking
MSC	Machine-side converter
ORB	Optimum relation-based
ORC	Optimal rotational cycle
OTC	Optimal torque control
P&O	Perturb and observe
PMSG	Permanent magnet synchronous generator
PSO	Particle swarm optimizer
PSF	Power signal feedback
RVS	Robust variable ste
SCIG	Squirrel-cage induction generator
SS	Small step
SOSMC	Second-order sliding mode controller
STA	Super-twisting algorithm
SVM	Support vector machine
SVPWM	Space vector pulse-width modulation
THD	Total harmonic distortion
VS	Variable step
VSWT	Variable-speed wind turbine
WECS	Wind-energy control system
WSE	Wind speed estimated
WT	Wind turbine

## Appendix A

**Table A1:** PMSG setting parameters.

Rated power	$P_{e}=10\;\mathrm{kw}$	Permanent magnet flux	$\psi_{m}=0.071\;\mathrm{wb}$
Stator resistance	$R_{s}=0.00829\;\Omega$	Number of pole pairs	$n_{p}=6$
Stator direct inductance	$L_{d}=0.174\;\mathrm{mH}$	Inertia	$J_{t}=0.089\;\mathrm{kg}\cdot m^{2}$
Stator quadrature inductance	$L_{q}=0.174\;\mathrm{mH}$	Friction	f=0.005N·m

**Table A2:** WT setting parameters.

Radius of the turbine	$R_{t}=2\;m$	Optimal tip speed ratio	$\lambda_{opti}=8.1$
Air density	$\rho =1.225\;\mathrm{kg}\cdot m^{3}$	power Coefficient	$C_{p}=0.48$
Pitch angle	$\beta =0^{{}^{\circ}}$		

**Table A3:** DC bus and grid setting parameters.

Grid resistance	$R_{g}=0.02\;\Omega$	Grid quadrature inductance	$L_{qg}=0.005\;H$
Grid direct inductance	$L_{dg}=0.005\;H$	DC-Link-Voltage	$V_{dc}=800\;v$
